# Reducing disparities in cardiovascular health in African Americans through integrated cardiovascular sleep care in outpatient setting

**DOI:** 10.1093/sleepadvances/zpac016

**Published:** 2022-05-12

**Authors:** William J Healy, Girardin Jean-Louis, Clyde W Yancy, Martha E Billings, Rami Khayat, Younghoon Kwon

**Affiliations:** Division of Pulmonary, Critical Care, and Sleep Medicine, Medical College of Georgia at Augusta University, Augusta, GA,USA; Departments of Psychiatry and Neurology, University of Miami, Miami, FL, USA; Department of Medicine/Division of Cardiology, Feinberg School of Medicine, Northwestern University, Chicago, IL,USA; Division of Pulmonary, Critical Care, and Sleep Medicine; University of Washington, Seattle, WA, USA; Division of Pulmonary and Critical Care Medicine, University of California-Irvine , Irvine, CA, USA; Division of Cardiology, University of Washington, Seattle, WA, USA

1) Cardiovascular health disparity in AA

Cardiovascular (CV) health disparity is a growing public health challenge. African American patients (AA) have a greater risk of CV morbidity and mortality than any other racial/ethnic groups in the United States [1]. In 2017, AA were more than twice as likely as non-Hispanic Asian or Pacific Islander persons to die of CV disease (CVD) [1]. Along with the known issue with access to healthcare, a key driver of the early onset and high burden of CVD in AA is the disproportionately high prevalence of CV risk factors in this population [2]. Therefore, one approach to improving CV health in AA should include early identification and modification of these CV risk factors that disproportionately affect AA and lead to development of CVD.

2) Impact of sleep on CV health and sleep health disparity in AA

An increasingly recognized contributor to CV risk in the population is sleep disorders and insufficient sleep [3]. Therefore, it is not surprising that sleep health is an emerging contributing factor to disparity in CVD outcomes in AA [4]. Among sleep disorders, obstructive sleep apnea (OSA), which affects over 26% of adults between the ages of 30 and 70 years, has been linked to an increased risk of hypertension, coronary artery disease, stroke, heart failure, and atrial fibrillation amongst others [5]. AA with OSA are at twice higher odds for having resistant hypertension than AA without OSA when adjusting for confounders [6].

Racial disparity in sleep health is increasingly being recognized [7]. AA are disproportionately affected by OSA. AA children are 4 to 6 times more likely to have OSA compared to white children [8]. More AA children than white children in this study had obesity, an important risk factor for OSA [8]. While AA have similar prevalence of OSA as whites (32% and 30% respectively) in adults, AA tend to have more severe OSA [9]. Moreover, AA have less acceptance of continuous positive airway pressure (CPAP), the first line therapy, than whites [10]. Beyond OSA, sleep time and circadian rhythm are critical contributor to CV health. A study of AA in Chicago, IL and surrounding suburbs showed that the average duration of sleep of AA was nearly 1 hr shorter than whites [11]. Shorter sleep duration affects quality of life, is associated with hypertension, and increases mortality [12]. Circadian misalignment has been shown to increase the presence of CV risk factors although the mechanisms are incompletely understood [13].

3) Main hypothesis: Disparity in sleep care is a contributor to CV health disparity

While complex socioeconomic, cultural, political, and behavioral factors contribute to CV health disparity, sleep health is an important aspect to address in any approach to addressing disparity in CV outcomes. We hypothesize that 1) sleep disparity is a potent mediator CV health disparity seen in AA. 2) Innovative approaches specifically tailored to addressing disparities in sleep care delivery will reduce sleep health disparity and thereby may improve CV health disparity [14].

4) Current status and major gap in sleep health delivery in cardiology clinics

Current models of delivering CV care are not equipped to address sleep health. The addition of a sleep health evaluation to the patient interview can add time constraints to cardiology clinic workflow. Therefore, sleep health, specifically OSA, is not adequately addressed especially if sleep specialists are not readily available for referral. We suspect that AA are less likely to be referred to a sleep specialist. This is likely tied to socioeconomic and educational disadvantages of AA as well as possible implicit bias of providers.

5) Innovative model incorporating sleep care into cardiology clinics (Sleep navigator) (future)

**Figure 1.**
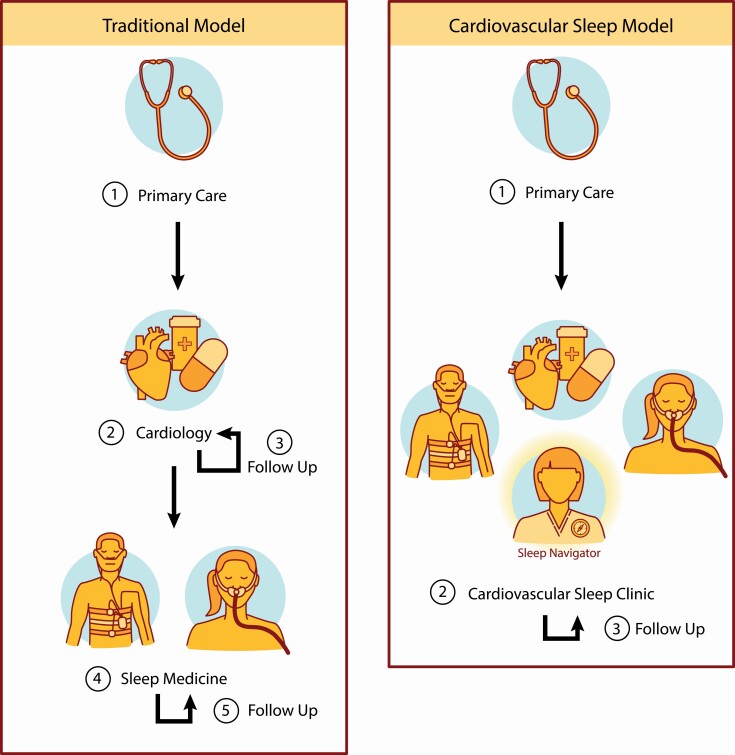
On the left, the current model of referrals to cardiology clinics and separate sleep medicine clinics is detailed. On the right, we propose an integrated model of cardiovascular sleep care leveraging a sleep navigator to streamline care. This is an example of one practical solution to disparities in sleep care although a much broader approach in addressing sleep health disparity is essential. Printed with permission from © 2021 Augusta University and Sarah H. Sutton.

To narrow this gap, we propose an integrated sleep care model within the cardiology clinic as illustrated in Figure 1. In the current model, the patient is burdened with multiple steps including additional referrals after the cardiology visit to address sleep needs. The proposed integrated CV sleep model is based on the premise that the evaluation of OSA is typically straightforward. Technological advances using digital health tools including currently validated screening instruments like STOP-BANG could help to capture risk level for sleep disorders and prompt sleep testing to be considered during the cardiology visit [15]. These data would be provided to a sleep navigator who has training in an allied health field (e.g., respiratory therapist, sleep technologist, or nurse) and practicing with support or supervision of a sleep specialist that can assist patients through testing and adhere to standing orders for common clinical scenarios. This leverages the advances in OSA screening technology including prompt diagnosis facilitated by home sleep apnea testing for most patients. Home tests can utilize semi-automated scoring which is manually edited with revised scoring by a board-certified sleep specialist. Rapid initiation of CPAP therapy using an autotitrating CPAP can obviate the need for a CPAP titration and save time in the initiation of therapy unless there are additional concerns. Both in-lab polysomnograms at a collaborating sleep laboratory or a home sleep apnea test can be obtained depending on sleep navigator provider review and co-morbidities. Indeed, what was once a tedious process of getting a patient initiated and followed on CPAP can now be streamlined with proper ancillary support, allowing cardiologists to be better positioned for a direct role in OSA care [16]. If a diagnosis of OSA is made, treatment can be initiated through streamlined orders and the follow-up care can be synchronized with the next cardiology visit. Furthermore, AA may benefit from implementation of telehealth sleep care that leverages technologies in screening, treatment, and coordination of care. With the additional clinic throughput that is possible with a sleep navigator, this position has the potential to pay for itself through increased clinical revenue and has overall health savings in terms of early diagnosis and treatment resulting in better overall outcomes.

This streamlined process will more benefit AA by reducing logistical challenges associated with traditional OSA care by reducing the amount of follow-up appointments. The sleep navigator would further focus on education and medical equipment issues to minimize additional burden on the busy cardiologist. When there are questions regarding treatment or deviations from protocol, or other sleep issues need to be addressed, the sleep navigator would engage a collaborating sleep specialist through telehealth. The sleep specialist would interpret sleep testing arising from the CV sleep clinic and would already have some insight into the patients’ sleep prior to any needed telehealth visits. Sleep navigator can also facilitate the inpatient referral process of patients with high risk for OSA to outpatient sleep evaluation. Identification and arrangement of sleep testing has been shown to improve outcomes in those adherent to CPAP therapy [14]. This approach can be particularly helpful to AA patients admitted to hospital.

Although we believe the sleep navigator will be particularly valuable to AA, it will benefit a larger cohort of patients seen in cardiology regardless of their background. While the sleep navigator would be one practical solution to improve the incorporation of sleep care into the cardiology practice, thereby addressing sleep disparity, improving sleep care in AA will be also empowered by an increase in the physician workforce with formal sleep medicine training. An important aspect of increasing workforce will be in recruiting under-represented minorities into the field of sleep medicine. The American Academy of Sleep Medicine (AASM) has started the AIRE program (Advancing Innovation in Residency Education) to pilot novel training pathways in sleep medicine to address the foreseeable shortage in the sleep medicine workforce. This includes blending training with other training programs in a competency-based curriculum along with also piloting part-time training pathways. We posit that patients could benefit from pathways that would increase the number of cardiologists that are board-certified in sleep medicine.

6) Addressing CV health disparity in AA through improving sleep health

The AA population is projected to increase by over 40% in the United States by 2060 necessitating urgent investigation into the role that sleep disorders play in the CV disparities in this group of patients [17]. A call to action from the Association of Black Cardiologists announced nearly a decade ago to improve disparities through increase awareness and interdisciplinary collaboration in OSA unfortunately has not made much progress [18]. Further, the NHLBI (National Heart, Lung, and Blood Institute) has recently prioritized improving sleep health in patients experiencing health disparities through a more patient-centered approach [19]. We posit an integrated model of CV sleep care along with expanding the sleep medicine physician workforce within the cardiology specialty will improve healthcare disparities in sleep delivery. In this regard, we welcome the recent establishment by the AASM of the Specialty Practice Accreditation program with cardiology practice [20]. We believe such a program is a crucial step in the conjoint efforts of reducing racial health disparities through sleep care in AA. We propose another call to action and invite all CV health as well as sleep society professionals to consider sleep health as an integral component to narrow the CV health disparity in AA.
